# Pcdh18a regulates endocytosis of E-cadherin during axial mesoderm development in zebrafish

**DOI:** 10.1007/s00418-020-01887-5

**Published:** 2020-06-01

**Authors:** Bernadett Bosze, Yosuke Ono, Benjamin Mattes, Claude Sinner, Victor Gourain, Thomas Thumberger, Sham Tlili, Joachim Wittbrodt, Timothy E. Saunders, Uwe Strähle, Alexander Schug, Steffen Scholpp

**Affiliations:** 1 Institute of Toxicology and Genetics, Karlsruhe Institute of Technology (KIT), 76021 Karlsruhe, Germany; 2 Living Systems Institute, School of Biosciences, College of Life and Environmental Sciences, University of Exeter, Exeter, EX4 4QD UK; 3 Steinbuch Centre for Computing, Karlsruhe Institute of Technology (KIT), Karlsruhe, 76021 Germany; 4 Department of Physics, Karlsruhe Institute of Technology (KIT), 76021 Karlsruhe, Germany; 5 Centre for Organismal Studies, Heidelberg University, 69120 Heidelberg, Germany; 6 Mechanobiology Institute, National University of Singapore, Singapore, 117411 Singapore

**Keywords:** Prechordal plate, Notochord, Migration, Endocytosis, Zebrafish

## Abstract

The notochord defines the axial structure of all vertebrates during development. Notogenesis is a result of major cell reorganization in the mesoderm, the convergence and the extension of the axial cells. However, it is currently not fully understood how these processes act together in a coordinated way during notochord formation. The prechordal plate is an actively migrating cell population in the central mesoderm anterior to the trailing notochordal plate cells. We show that prechordal plate cells express Protocadherin 18a (Pcdh18a), a member of the cadherin superfamily. We find that Pcdh18a-mediated recycling of E-cadherin adhesion complexes transforms prechordal plate cells into a cohesive and fast migrating cell group. In turn, the prechordal plate cells subsequently instruct the trailing mesoderm. We simulated cell migration during early mesoderm formation using a lattice-based mathematical framework and predicted that the requirement for an anterior, local motile cell cluster could guide the intercalation and extension of the posterior, axial cells. Indeed, a grafting experiment validated the prediction and local Pcdh18a expression induced an ectopic prechordal plate-like cell group migrating towards the animal pole. Our findings indicate that the Pcdh18a is important for prechordal plate formation, which influences the trailing mesodermal cell sheet by orchestrating the morphogenesis of the notochord.

**Electronic supplementary material:**

The online version of this article (10.1007/s00418-020-01887-5) contains supplementary material, which is available to authorized users.

## Introduction

The notochord is the most prominent hallmark of the phylum Chordata and serves as the common embryonic midline structure for all their members, including humans. Serving as an embryonic scaffold for the surrounding mesoderm to subsequently form the skull, the membranes of the brain, and, most importantly, the vertebral column (Stemple 2005), it plays a central role in the genesis of the vertebral body. Generation of the dorsal mesoderm, including the notochord, is based on immense cellular rearrangements and the zebrafish has been proven to be a suitable in vivo system to study this process. During zebrafish embryogenesis, the notochord originates from the dorsally localized shield organizer of the embryos when cells break their cell–cell junctions and undergo single-cell ingression at the onset of gastrulation (Warga and Kimmel 1990). After internalization, these mesodermal progenitor cells organize into a coherent cell sheet to migrate from the embryonic margin towards the animal pole of the embryo. This cell sheet can be subdivided into the prechordal plate (ppl) mesoderm, the axial notochordal plate, and the surrounding lateral plate mesoderm (lpm, Kimelman and Griffin 2000). Subsequently, the notochordal plate narrows and elongates in the perpendicular axis to finally transform into the notochord, whereas the lpm cells migrate towards the midline to form the remaining mesodermal organs. The molecular mechanisms controlling these cell movements, generally known as convergence and extension, have been extensively studied in the lpm (Roszko et al. 2009; Myers et al. 2002). Wnt-planar cell polarity (PCP) signalling modulates E-cadherin cell adhesion junctions, which mediate collective cell polarization to coordinate cell movements (Theveneau and Mayor 2012; Heisenberg and Solnica-Krezel 2008; Heisenberg et al 2000). Directed migration is a prerequisite for coordinated cohort movement, and polarized distribution of Wnt-PCP components has been suggested to be crucial for this process in the zebrafish lpm (Heisenberg et al. 2000; Ulrich et al. 2003; Roszko et al. 2015). Therefore, a hypothesis for notochord formation suggests that, similar to the lpm, E-cadherin-mediated cell migration leads to the intercalation and elongation of the notochord plate cells to form the rod-shaped notochord (Tada and Heisenberg 2012; Feldman et al. 1998); however, the molecular mechanism is less clear.

Protocadherins represent a large family of non-classical cadherins (Hayashi and Takeichi 2015). Protocadherins together with cadherins have been suggested to influence cell adhesion; for example, protocadherin 8 (PAPC) mediates sorting of paraxial mesoderm from axial mesoderm (Kim et al. 1998; Medina et al. 2004) in concert with C-cadherin during *Xenopus* gastrulation (Chen and Gumbiner 2006). Recently, the function of protocadherin 18a (Pcdh18a) has been described in zebrafish suggesting that Pcdh18a regulates cell adhesion during gastrulation and cell movements in embryonic patterning (Aamar and Dawid 2008).

Here, we describe the expression of Pcdh18a in cells in the ppl. We show that Pcdh18a regulates endocytosis of E-cadherin (E-cad). Pcdh18a-mediated E-cad endocytosis allows ppl cells to cluster and migrate faster compared to the surrounding lpm and influences the trailing axial mesoderm and its transformation into the notochord.

## Materials and methods

### Zebrafish husbandry

Adult zebrafish (*Danio rerio*) were maintained at 28.5 °C on a 14 h light/10 h dark cycle (Brand et al. 2002). The data we present in this study were acquired from an analysis of wild-type zebrafish (AB) and transgenic zebrafish lines: *Tg(-1.8 gsc:GFP)ml1* (Dumortier et al. 2012), *Tg(-2.2shha-:GFP:ABC)sb15* (Shkumatava et al. 2004), *Tg(h2afx:EGFP-rab5c)mw5*, *Tg(h2afx:EGFP-rab7)mw7* and *Tg(h2afx:EGFP-rab11a)mw6* (Clark et al. 2011). All animal work was approved by the GM safety committee of the University of Exeter and the Home Office by appropriate licences as specified in the following: S Scholpp’s project licence No P5FA1DA44, Use of Animals (Scientific Procedures) Act ASPA 1986, June 2017–June 2022 and the personal licences awarded to S Scholpp (IC325CEA2) and Y Ono (IE1B962B6).

### Functional analysis

Transient knockdown of gene expression was performed using Morpholino oligonucleotides (MO). The following antisense oligomers were used:

control MO 5′-CGAAGTCTACGTCGGAATGCAGG-3′ (Mattes et al. 2012);

Pcdh18a UTR MO 5′-TCCGTCAGGCACTGCAAAAATATAC-3′ (Aamar and Dawid 2008);

Pcdh18a translation blocking MO 5′-ACCCTTGCTAGTCTCCATGTTGGGC-3′ (Aamar and Dawid 2008).

Pcdh18a function was inhibited by injecting a 0.5 mM concentration of each Morpholino oligomer at the one-cell stage. The following plasmids were used for the overexpression studies: Pcdh18a in pCS2+, Pcdh18a-GFP in pCS2+, Pcdh18a-mCherry in pCS2+, glycosylphosphatidylinositol-anchored mCherry (memCherry) in pCS2+, GAP43-GFP, and E-cadherin-GFP and E-cadherin-mCherry (gift from Erez Raz, University of Münster). Capped and in vitro transcribed mRNAs (mMessage Machine Kit, Ambion) were microinjected into one-cell stage embryos.

### Generation of *pcdh18a* mutant line

Single guide RNAs (sgRNAs) to target the *pcdh18a* locus in CRISPR/Cas9 mutagenesis were designed and evaluated for potential off-target sites using CCTop online predictor (Stemmer et al. 2015; https://crispr.cos.uni-heidelberg.de/), selecting the target sequence 5′-CAGAGCAAGTTTGAGTAAAGTGG-3′. For sgRNA assembly, a pair of synthesized oligomers (5′-TAGGGAGCAAGTTTGAGTAAAG-3′; 5′-AAACCTTTACTCAAACTTGCTC-3′) was annealed, ligated into the DR274 (Addgene plasmid #42250) vector (Hwang et al. 2013), linearized with FastDigest Eco31l (Thermo Fisher Scientific), and used for in vitro transcription with the T7 MegaShortScript Kit (Ambion). The Cas9 protein was purchased from Thermo Fisher Scientific (B25640). Prior to the injection, the Cas9 protein and sgRNA were diluted in RNAse-free water and incubated for 5 min at RT for complex formation (Burger et al. 2016). For the identification the following primers were used: pcdh18a for: TGGCACTAAAGGAGGCTTTG; pcd18 rev mut/WT: CACTTTACTCAAACTTGCTCTGC; pcdh18a rev ctrl: ACCAGGATGGAGAGATCAGC.

### Compounds and inhibitors

Dechorionated embryos from the sphere stage to 80% epiboly were treated with 30 µM SB-505124 (Sigma) to block mesoderm formation. In cell culture, 1 µM Dyngo-4a (Tocris) was used to block clathrin-mediated endocytosis.

### Cell culture experiments

HeLa cells were obtained from Sigma. L cells that were stably transfected with human E-cadherin-GFP (E-cad-GFP+ L cells) were provided by Clemens Franz (KIT; Fichtner et al. 2014). Both cell types were cultured in high-glucose DMEM, supplemented with 10% FBS and 1% Pen/Strep. The cells were transfected using the FuGene HD Transfection Reagent (Invitrogen) at 80% confluence.

### Wound-healing assay

Wound-healing assays were performed using cell culture inserts (IBIDI). Approximately, 5 × 100 cells were cultured in each cell culture reservoir, which was separated by a 500 µm-thick wall. After 6 h of cultivation, the culture inserts were removed and cell migration was monitored for several hours using an Axiovert 800 M inverted microscope. The obtained time-lapse images were analysed using ImageJ software (National Institutes of Health). To block E-cadherin-mediated adhesion by adherens junctions, E-cad-GFP+ L cells were treated with 50 mg/ml of the anti-E-cad antibody DECMA-1 (U3254, Sigma Aldrich) during the wound-healing assay.

### Western blotting

For the Western blots, whole-cell extracts and whole embryo extracts were prepared and resolved by 2–10% gradient SDS-PAGE. The proteins were then transferred to a PVDF membrane. The membrane was incubated with anti-GFP (Sigma, 1:1000) and anti-PCNA (Abcam, 1:5000) primary antibodies for 4 h at room temperature. A secondary antibody rabbit anti-mouse IgG HRP (abcam, 1:1000) was used for detection.

### Fluorescence recovery after bleaching (FRAP) assay

For the FRAP assay, four embryos were micro-injected with either E-cad-GFP (100 ng mRNA), with E-cad-GFP (100 ng mRNA)/Pcdh18a-mCherry (200 ng mRNA), or with E-cad-GFP (100 ng mRNA)/Pcdh18a ECD (200 ng mRNA). After 5 hpf, embryos were mounted in agarose. A Nikon A1 Confocal Laser Microscope with a blue diode laser (405 nm) was used to perform the FRAP experiments. A laser light (405 nm) bleached 3 μm spots on the cell membrane. The fluorescence within three independent bleached spots per embryo were tracked by confocal microscopy before and after bleaching in 10 s intervals. As E-cad-GFP moves into the bleached area, fluorescence recovers with an exponential time course. Within the spot, pre-bleaching fluorescence was set to 1 and post-bleaching fluorescence to 0.

### Three-dimensional optic flow tissue analysis

We used a custom three-dimensional optic flow code written in Matlab to measure the three-dimensional velocity fields in both the ectoderm and mesoderm tissues. The memCherry expression in the ectoderm was used to calculate ectoderm velocity and the *gsc:GFP* expression in the axial mesoderm to calculate mesoderm velocity. To avoid overlap between the two tissues velocity fields, we manually masked the ectoderm for mesoderm measurements and the mesoderm for ectoderm measurements. A sliding box of 20 × 20 × 8 pixels was used to calculate the local velocity field in each voxel.

### Deep RNA sequencing and data analysis

Wild-type embryos were microinjected with 200 ng of Pcdh18a mRNA or 0.5 mM Pcdh18a MO. At 24 hpf, pools of 50 embryos from the wild-type and the injected clutches were collected. Total RNA extraction was performed with TRIzol (Invitrogen) according to the manufacturer’s protocol. The extracted total RNA samples were tested on RNA nanochips (Bioanalyzer 2100, Agilent) for degradation. Sequencing libraries were generated with the TruSeq mRNA kit v.2 (Illumina). The size and concentration of the sequencing libraries were determined with DNA-chip (Bioanalyzer 2100, Agilent). Multiplexed samples were loaded on a total of six sequencing lanes. Paired end reads (2 × 50 nucleotides) were obtained on a Hiseq1000 using SBS v3 kits (Illumina). The sequencing resulted in 300 million pairs of 50-nucleotide-long reads. The reads were mapped against the zebrafish genome (Zv9) using TopHat version 1.4.1. Gene expression was determined with HTSeq version 0.5.3p3.

### Embryological manipulation assay

Donor embryos were microinjected with 0.5 mM control MO or Pcdh18a MO mixed with a lineage marker (miniEmerald, Thermo Fisher) at the one-cell stage. The host embryos were wild-type embryos or embryos that had been injected with Pcdh18a MO at the one-cell stage. At the shield stage, 50 cells were removed from each donor embryo with a needle and injected into the centre of the shield mesoderm of the host embryos. The host embryos were then allowed to develop until 90% epiboly (9 hpf) and fixed for in situ hybridization. In vivo two-photon laser-targeted ablation of individual cell rows in the axial mesoderm was performed with a Leica Sp2. At 7 hpf, *Tg(gsc:GFP)* embryos were mounted dorsal side up and ultrashort laser pulses were used to ablate the 5th GFP-positive cell row or the 15th GFP-positive cell row. The embryos were raised until 10 hpf for fluorescence imaging and subjected to in situ hybridization.

### In situ hybridization (ISH)

Prior to staining, embryos at the desired stage were fixed in 4% paraformaldehyde/PBS overnight at 4 °C. Whole-mount ISH was performed as previously described (Scholpp and Brand 2003). Antisense RNA probes against *ntl*, *hgg*, *pcdh18a*, *gsc*, *wnt11*, *dkk*, *chordin*, *snail1a*, *snail1b* and *e-cadherin (cdh1)* were used.

### Live embryo imaging and image analysis

Confocal image stacks were obtained using the Leica TCS SP5 X confocal laser-scanning microscope. We collected a series of optical planes (z-stacks) to reconstruct the imaged area. The step size of the acquired z-stack was 1 µm and was chosen based on the optimal z-resolution of the 63 × objective with a numerical aperture of 0.9. The images were further processed using Imaris software 7.5 (Bitplane AG). Cell shape (roundness) was measured using ImageJ software and approximately 100 cells from each group were analysed.

### Hamiltonian for cellular Potts model (CPM)

1 $$\begin{aligned} H &= \sum\limits_{{{\text{cells}}}} {\sum\limits_{{{\text{neighbours}}}} {J_{{\tau \left( {\sigma \left( {\vec{x}} \right)} \right)\tau \left( {\sigma \left( {\vec{x^{\prime}}} \right)} \right)}} } } \left( {1 - \delta _{{\sigma \left( {\vec{x}} \right)\sigma \left( {\vec{x^{\prime}}} \right)}} } \right) \hfill \\ \quad \quad &+ \sum\limits_{{{\text{cells}}}} {\lambda _{V} \left( {V\left( \sigma \right) - V_{0} \left( \sigma \right)} \right)^{2} } \hfill \\ \quad \quad &+ \sum\limits_{{{\text{cells}}}} {\lambda _{S} \left( {S\left( \sigma \right) - S_{0} \left( \sigma \right)} \right)^{2} } . \hfill \\ \end{aligned}$$

2 $$\Delta H_{M} = \mu \left( {\tau \left( {\vec{x^{\prime}}} \right)} \right)\lambda _{M} \left[ {c\left( {\vec{x^{\prime}}} \right) - c\left( {\vec{x}} \right)} \right].$$

3 $$\Delta H = \Delta H_{B} + \Delta H_{M} .$$

4 $$P\left( {\gamma \to \gamma ^{\prime}} \right) = \left\{ \begin{gathered} e^{{ - \tfrac{{\Delta H}}{T}}} ,\;{\text{if}}\;\Delta H_{{{\text{Tot}}}} \ge 0, \hfill \\ 1,\quad \;{\kern 1pt} {\kern 1pt} {\kern 1pt}\,\, {\text{if}}\;\Delta H_{{{\text{Tot}}}} {\text{ < }}0. \hfill \\ \end{gathered} \right.$$

The definition for the Hamiltonian *H* and modifications to *ΔH* for our CPM are shown. Exact parameter values are found in Supplementary Fig. S6h. (1) The Hamiltonian for state *γ* consists of three sums and defines the total energy of the system. The first sum runs over each cell *σ* at a lattice site $\overrightarrow{x}$ and its neighbouring lattice sites $\vec{x^{\prime}}$. This sum represents the energetic contributions by the cellular adhesiveness *J*, which is dependent on the cellular types $\tau \left( {\sigma \left( {\vec{x}} \right)} \right)$ and $\tau \left( {\sigma \left( {\vec{x^{\prime}}} \right)} \right)$. For the exact values of *J*, see Supplementary Fig. S6g. The Kronecker-delta *δ* makes sure that only different cells contribute and self-interactions are excluded. The second and third sums run over each cell and sum up volume and surface contributions scaled by a factor *λ*_*V*_, respectively *λ*_*S*_. Each cell tries to preserve its original volume *V*_0_ and surface *S*_0_. (2) We defined a modification ΔHM to introduce mobility into our simulations with a linear anterior–posterior potential *c*. This modification is coupled to the mobility *µ* of the source cell at the neighbouring lattice site $\vec{x^{\prime}}$ for the hypothetical state *γ*' and the mobility constant *λ*_*M*_. See Supplementary Fig. S6a for a visual explanation of the simulation scheme. The total change in *ΔH* is obtained from both contributions of *ΔH*_*B*_ = *H*(*γ*') − *H*(*γ*) and *ΔH*_*M*_. (3). The hypothetical state *γ*' is accepted with a probability as given by the Metropolis criterion.

## Results

### Prechordal plate cells are required to shape the zebrafish notochord

**Fig. 1:**
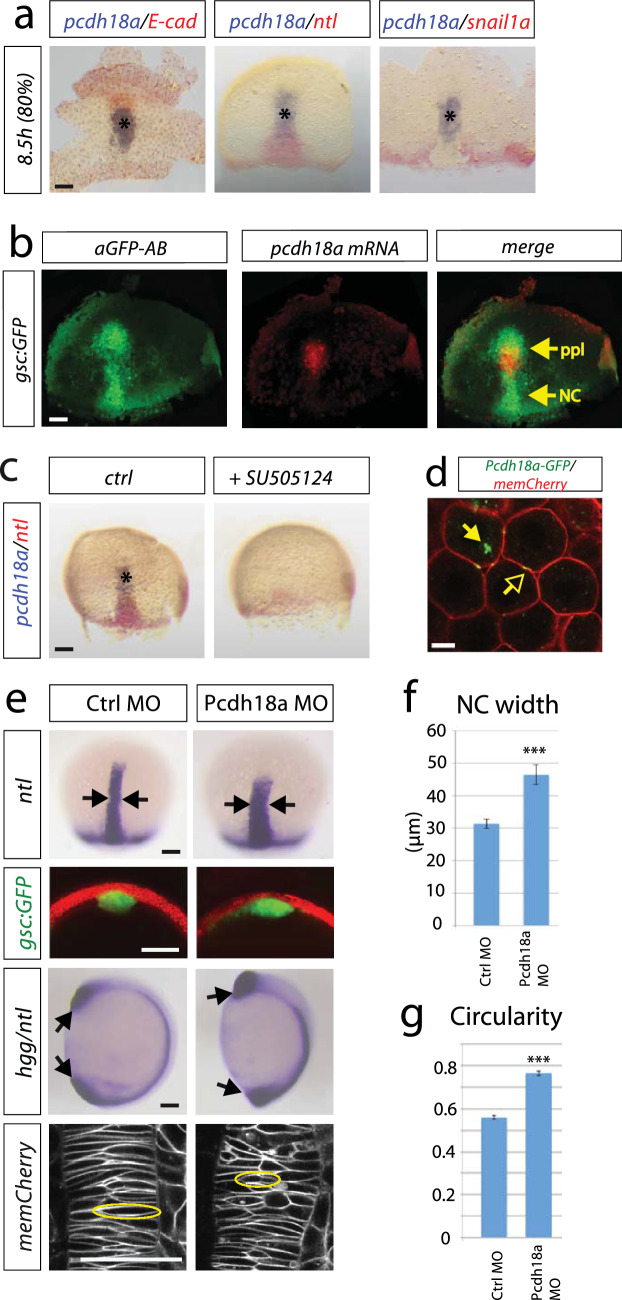
A cell group in the axial mesoderm regulates notochord morphogenesis in zebrafish. **a** Characterization of the prechordal plate (ppl) by whole-mount double in situ hybridization (ISH) of wild-type (WT) zebrafish embryos with the indicated markers. Scale bar: 100 µm. **b** Mapping of *pcdh18a* mRNA expression (red) relative to *Tg(gsc:GFP)* expression labelled by anti-GFP antibody (green). Notably, *gsc:GFP* labels the prechordal plate (ppl) and the trailing notochord (NC). Scale bar: 100 µm. **c** Inhibition of nodal signalling by SB505124 treatment (30 µM) from 4 to 8.5 h post-fertilization (hpf). Scale bar: 100 µm. **d** Confocal image of live zebrafish embryo at 5 hpf showing subcellular localization of Pcdh18a-GFP. Glycosylphosphatidylinositol-anchored mCherry (memCherry) marks cell membranes. Arrows indicate punctae of Pcdh18a localization at the membrane (open arrows) and intracellularly (closed arrows). Scale bar: 10 µm. **e** WT embryos or *Tg(gsc:GFP)* embryos were injected with Pcdh18a MO (0.5 mM). Morpholino-based knockdown of Pcdh18a leads to a wider and shorter notochord marked by *ntl* expression at 9 hpf (arrows). See Supplementary Fig. S1h for control experiments. Analysis of the shape of the notochord in a cross section of *gsc:GFP* transgenic embryos that were injected with the indicated constructs at 10 hpf. At 11 hpf, the body length was significantly shorter in the Pcdh18a-deficient embryos, as shown in an ISH-based analysis of notochord *hgg/ntl* (*n* = 20/32, arrows). Confocal microscopy-based analysis of cell shapes in the notochord of embryos that were microinjected with the indicated constructs at 12 hpf (cells with exemplary morphology were surrounded with a yellow circle). Scale bar: 100 µm. **f** *pcdh18a* MO-injected embryos show a wider axial mesoderm compared to Ctrl MO injected embryos (four embryos each). **g** Circularity of NC cells was measured in total of 1000 cells in five different embryos each. A circle has a circularity of 1.0, while noncircular shapes have a lower value of circularity. The error bars represent the SEM and significance as indicated (***p value < 0.001; unpaired Student’s *t* test)

We mapped the expression of key cell adhesion molecules relative to the expression of mesodermal marker genes to characterize the axial mesoderm during early notochord development in zebrafish. In the central mesoderm, we identified a cell cluster that expresses *pcdh18a* and *e-cadherin* (*e-cad*, Fig. 1a; Supplementary Fig. S1a–g). This cell cluster is located in the ppl anterior to the notochord labelled by *ntl* and *gsc:GFP* expression (Fig. 1a, b; Supplementary Fig. S1). Nodal signalling is required for the induction and involution of the mesoderm-derived notochord at the shield organizer (Feldman et al. 1998; Sampath et al. 1998; Schier et al. 1997). Blocking the nodal-signalling cascade lead to a lack of the *pcdh18a*-positive ppl cells as well as the *ntl*-positive notochord, confirming the observation that *pcdh18a* is expressed in the mesodermal plate (Fig. 1c). Protocadherins are transmembrane proteins and, in concert, we find Pcdh18a clusters at the plasma membrane and in intracellular vesicles (Fig. 1d).

To analyse whether Pcdh18a is required for mesoderm organization in the gastrula, we altered the Pcdh18a levels by using a Morpholino (MO)-based antisense approach with two independent Morpholino oligomers targeting the 5′UTR and the translation start site of *pcdh18a* (Aamar and Dawid 2008; Supplementary Fig. S1h). We found that a knock-down of *pcdh18a* expression led to a wider axial mesoderm, as shown by the lateral expansion of the *ntl* expression domain and of the *gsc* domain in *Tg(gsc:GFP)* zebrafish embryos (Fig. 1e, f; Supplementary Fig. S1i). At 26 h post-fertilization (hpf), the head and trunk display a wild-type (WT) phenotype; however, the tail shows a strong deformation (Supplementary Fig. S1j). During notogenesis, central mesodermal cells take on a bipolar shape and intercalate mediolaterally and pull together (convergence; Shih and Keller 1992). In zebrafish, polarized radial intercalations of axial mesoderm separates anterior and posterior neighbours, lengthening the field along the AP axis (extension; Tada and Heisenberg 2012; Feldman et al. 1998). Therefore, we investigated the shape of the notochord cells in Pcdh18a-deficient embryos at 12 hpf (six-somite stage). We found that the cells had a less bipolar shape and displayed a more circular form in embryos with reduced Pcdh18a levels (Fig. 1e, g), suggesting that mediolateral narrowing and AP lengthening of axial mesoderm are reduced. Next, we generated a genetic mutant using the CRISPR/Cas9 system and isolated a mutant allele which has a 5 bp deletion in exon1 of the *pcdh18a* gene (Supplementary Fig. S2a). However, both zygotic and maternal zygotic (MZ) *pcdh18a* mutant embryos did not exhibit the phenotypes observed in MO-injected embryos (Supplementary Fig. S2b–d). Next, we microinjected the Morpholino oligomers in the MZ mutant background, and similarly the shape of the notochord was not altered, suggesting that the loss of Pcdh18a function in the mutants was rescued by a genetic compensation mechanism (Rossi et al. 2015; El-Brolosy and Stainier 2017; El-Brolosy et al. 2019). Therefore, we used a Morpholino-based knock-down approach in the following functional analysis.

**Fig. 2:**
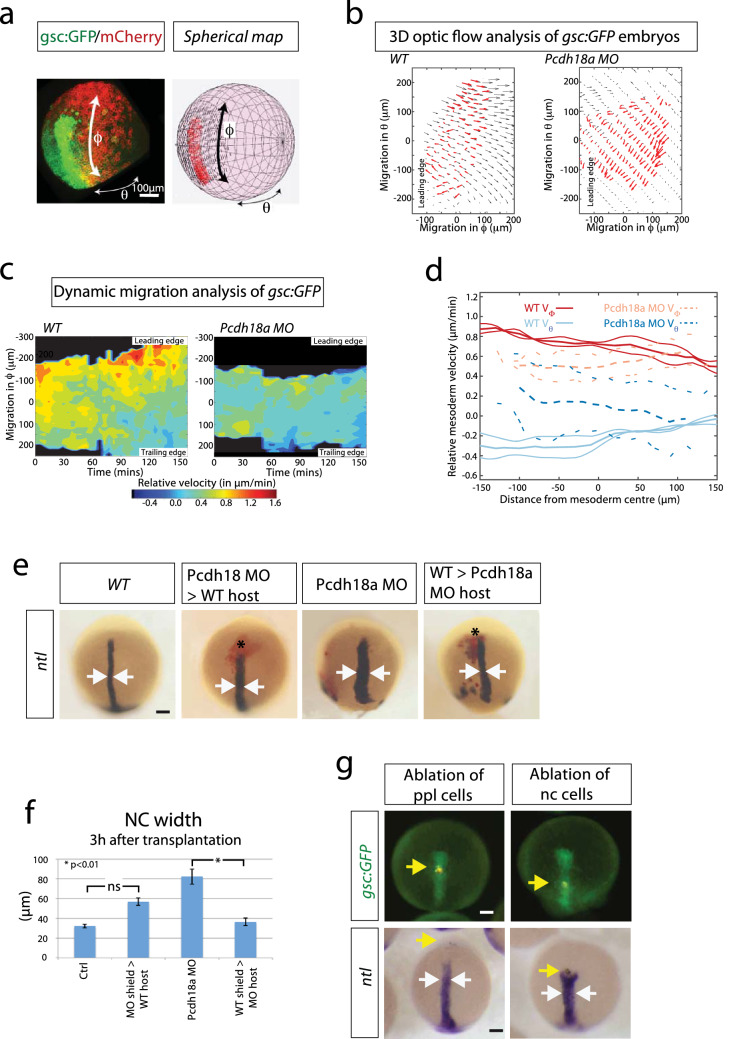
Analysis of the influence of the ppl on notochord morphogenesis. **a**–**d** 3D-optic flow analysis. **a** Spherical mapping of the optic flow of the fluorescent signal of a *gsc:GFP*/memCherry embryo onto a spherical coordinate system with *θ* as azimuth angle and *ϕ* as polar angle. **b** 2D Mercator projection of flow field of mesoderm (red arrows) compared with ectoderm above (black arrows). Both embryos analysed at 5 hpf. **c** The corresponding heat map kymograph shows the relative velocity of the mesoderm roughly every 5 min. **d** Thick solid (dashed) lines are smoothed average profiles of wild-type (WT; Pcdh18a deficient) embryos, with individual embryo profiles shown in lighter lines in *ϕ* (red/orange) and *θ* (light/dark blue) directions. **e** *pcdh18a* MO donor shield was transplanted into WT hosts and vice versa. After 3 h, the embryos were fixed and subjected to ISH against *ntl*. Donor cells are marked in red. Arrows indicate the width of the notochord. **f** Quantifications display mean value, standard error of mean (SEM), and significance level of six independent embryos per experiment as indicated (*p value < 0.01; unpaired Student’s *t* test). **g** Ablation of cell rows in the ppl (5th GFP positive cell row) or at the ppl-notochord border (15th GFP positive cell row) in the *Tg(gsc:GFP)* fish line. Embryos were injected with a nuclear marker (Histone 2B-mCherry) and cell rows were ablated at 7 hpf using ultrashort laser pulses of a two-photon microscope. Embryos were raised to 10 hpf, fixed, and subjected to ISH against *ntl*. After ablation of a cell row in the ppl, embryos develop an elongated notochord (*n* = 11/11), whereas the notochord progenitor cells move slower and a gap appears towards the ppl in embryos with ablation of a cell row at the ppl–notochord border. Consequently, the trailing *ntl* expression domain remains shorter and broader (*n* = 6/10, white arrows). Yellow arrows mark the ablated cell rows. Scale bar: 100 µm

Next, we investigated the migratory properties of the central mesoderm. We performed a three-dimensional optic flow analysis, 3D-KLT (Vig et al. 2016), in zebrafish embryos to obtain local tissue velocity measurements of both the mesoderm and ectoderm (see “Materials and Methods” for details). To aid quantification, we mapped the flow of the migrating tissues onto a sphere (obtained from a spherical fit of the embryo shape) and calculated velocities in spherical coordinates. The sphere’s equator and poles were oriented such that the main axis of elongation of the mesoderm was aligned with the meridian (Fig. 2a). For the flow analysis of the forming notochord, we used time-lapse movies from *Tg(gsc:GFP)* embryos injected with memCherry and scanned from 5 to 8 hpf (Fig. 2a). In this way, we calculated the velocity field for the *gsc:GFP*-positive axial mesoderm and the ectoderm above in WT and *pcdh18a* morphant embryos (Fig. 2b; Supplementary Fig. S3a). From these, we generated kymographs of the relative velocity between the mesoderm and ectoderm (Fig. 2c; Supplementary Fig. S3b). We found that in WT embryos, the leading edge and trailing edge of the ppl have distinct dynamics with respect to the overlying ectoderm: the leading cell population migrates faster (red-yellow areas) towards the animal pole compared to the trailing notochordal plate (green-blue areas) in the *ϕ* direction (Fig. 2c). The drop in relative migration speed from animal to vegetal becomes more apparent over time (after 150 min). Next, we compared the mesoderm dynamics in WT embryos with Pcdh18a-deficient embryos. We found that the ppl cells migrate slower in the *ϕ* direction in Pcdh18a-deficient embryos (Fig. 2c, d). In contrast, we found that migration in the *θ* direction is enhanced in the ppl cells of morphants (Fig. 2d; Supplementary Fig. S3c, d), whereas it is reduced in the trailing edge (Supplementary Fig. S3c, d). We hypothesized that pronounced *ϕ* migration mediated by Pcdh18a function in the ppl contributes to enhanced convergence (*θ* migration) of the follower cells—the notochordal plate.

To test whether Pcdh18a-positive ppl cells influence the organization of the trailing notochord, we altered the Pcdh18a levels in the ppl in a cell transplantation experiment (Fig. 2e, f). We discovered that the notochord of host embryos carrying a Pcdh18a-deficient ppl is wider than the control embryos supporting our previous results (Fig. 1e). In a reverse experiment, we introduced the ppl of WT origin into Pcdh18a-deficient embryos. The axis of *pcdh18a* morphant embryos with WT ppl was significantly thinner compared to the *pcdh18a* morphant embryos. Conversely, WT embryos transplanted with Pcdh18a-deficient ppl showed a slightly wider notochord compared to control embryos. Next, we performed a pulsed laser ablation experiment in the *Tg(gsc:GFP)* embryos, in which we ablated a cell row in the ppl and one in the anterior notochord. Ablation of a cell row in the ppl did not lead to an obvious alteration in the morphology of the chordamesoderm (Fig. 2g). However, ablation of a cell row in the anterior notochord led to the formation of a gap between the ppl and the notochordal plate, and these embryos displayed a shorter and wider notochord. These data suggest that the ppl influences the notochord morphogenesis. In summary, these data suggest that the Pcdh18a-positive ppl constitutes a cell cluster which organizes the elongation and intercalation of the posteriorly trailing notochord.

### Pcdh18a regulates E-cad endocytic recycling

**Fig. 3:**
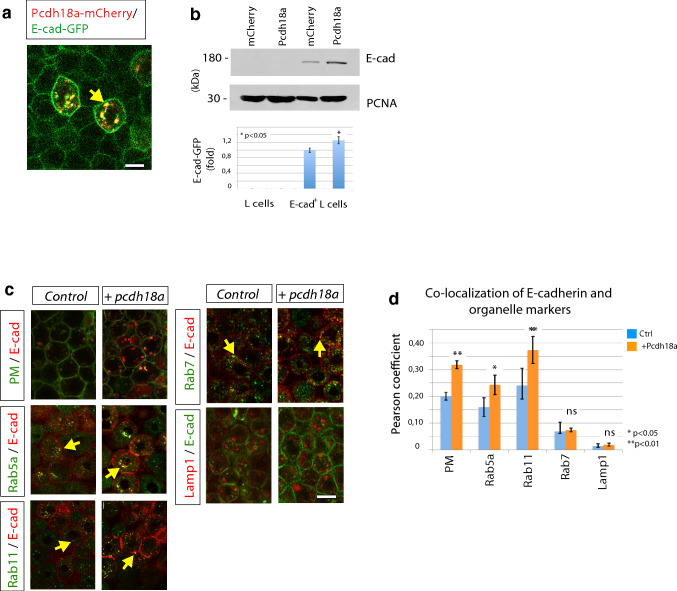
Pcdh18a regulates recycling of the E-cadherin. **a** Confocal image of zebrafish embryo at 5 hpf. Embryos were microinjected with 0.1 ng of mRNA for the indicated constructs and were imaged in vivo at 5 hpf. Pcdh18a is localized in the cell membrane and in endocytic vesicles, together with E-cadherin (E-cad). **b** Quantification of the E-cad levels in the Pcdh18a-transfected L cells. Equivalent amounts of lysates from murine L cells or stably E-cadherin-GFP-transfected L cells that had been transfected with Pcdh18a were Western blotted and probed with an anti-GFP antibody; the results showed a 26% increase in the E-cadherin-GFP protein levels after Pcdh18a transfection. The sample blot shows different parts of the same blot and PCNA was used as a loading control. The experiments were performed in independent triplicate (*p value < 0.05; unpaired Student’s *t* test). **c** Endocytic routing of E-cad at 50% epiboly. WT embryos and *Tg(rab5-GFP)*, *Tg(rab7-GFP)*, and *Tg(rab11-GFP)* stable transgenic embryos were microinjected with 0.1 ng of the mRNAs for the indicated constructs. Arrows indicate E-cad localization with Rab proteins and Lamp1-positive vesicles. **d** Pearson’s co-localization coefficient was calculated from 70 µm thick confocal stacks of five different embryos, each from **c**. The error bars represent the SEM and significance, as indicated (*p value < 0.05, **p value < 0.01; unpaired Student’s *t* test). Scale bar: 10 µm

Cadherins accumulate at cell–cell contact sites, such as adherens junctions, to regulate cell migration and cell adhesion (Hayashi and Takeichi 2015), similar to protocadherins, which influence homophilic and heterophilic interactions of cadherins (Hayashi et al. 2014; Brasch et al. 2012). We therefore sought to determine a possible interaction between the identified ppl-transmembrane proteins: Pcdh18a and E-cad. Co-localization of Pcdh18a-mCherry with E-cad-GFP was observed in zebrafish gastrula and in mouse fibroblasts that stably express E-cad-GFP (E-cad-GFP+ L cells) (Fig. 3a; Supplementary Fig. S4a, b). Remarkably, we observed increased E-cad-GFP fluorescence in vesicles and in the membrane in zebrafish blastula cells that co-express Pcdh18a. A Western blot analysis of stably transfected E-cad-GFP+ L cells also revealed an increase in E-cad expression in cells that co-express Pcdh18a (Fig. 3b). To determine which Pcdh18a-dependent mechanism might stabilize E-cad levels, as Pcdh18a function is dispensable for E-cad expression (Supplementary Fig. S7b), we tested whether Pcdh18a affects the subcellular routing of E-cad (Fig. 3c). We detected increased localization of E-cad in the plasma membrane, in Rab5a-positive early endosomes, and in Rab11-positive recycling endosomes when Pcdh18a was co-expressed (Fig. 3d). However, co-localization changes with Rab7-positive late endosomes and Lamp1-positive lysosomes was below detection level. These experiments suggest that Pcdh18a more effectively guides E-cad to the recycling pathway, hence enhancing the E-cad protein levels.

**Fig. 4:**
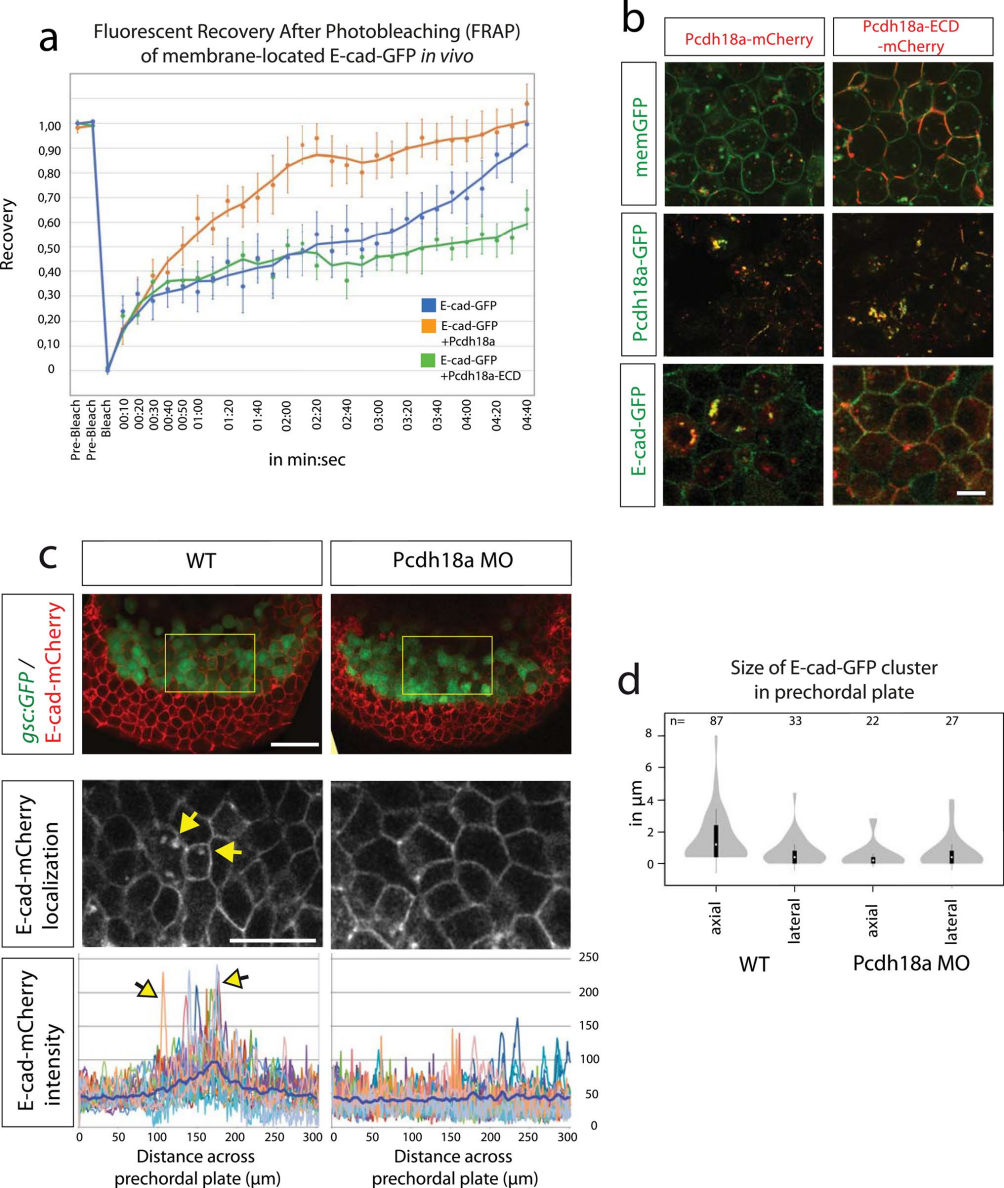
Pcdh18a domains and their importance with regard to endocytosis. **a** After photobleaching of a 3 µm spot at the cell membrane of E-cadherin-expressing embryos, new E-cadherin-GFP molecules moved into the bleached area from adjacent membrane regions, resulting in a return of 90% of fluorescence within 4 min 20 s (blue). Co-expression of Pcdh18a increased the speed of recovery and a 90% recovery was reached after 2 min 20 s (orange). Co-expression of Pcdh18a-ECD diminished FRAP of E-cadherin-GFP (green). Moving-average trendline was calculated with period 3. **b** Confocal images of zebrafish embryos at 5 hpf (50% epiboly). Embryos were injected with 0.1 ng mRNA of indicated constructs. In the deletion construct Pcdh18a-ECD, the intracellular domain was replaced by a mCherry domain. Pcdh18a-mCherry was localized to vesicles, whereas Pcdh18a-ECD-mCherrry was strongly localized to the cell membranes. Pcdh18a-GFP/Pcdh18a-mCherry and Pcdh18a-GFP/Pcdh18a-ECD-mCherry showed co-localization at the membrane and in vesicles suggesting homophilic interaction. Pcdh18a-mCherry/E-cadherin-GFP suggest heterophilic interaction. Pcdh18a-ECD-mCherry and E-cadherin-GFP were observed mainly at the membrane and did not co-localize suggesting that the intracellular domain of Pcdh18a is required for interaction and co-internalization with E-cad. Scale bar: 10 µm. **c** *Tg(gsc:GFP)* embryos were injected with 0.1 ng of the e-cadherin-mCherry mRNA or co-injected with the *pcdh18a* MO (0.5 mM) and subjected to confocal microscopy analysis at 8 hpf. A cross section at the level of the ppl reveals enhanced E-cad localization at the plasma membrane and in endocytic vesicles, as shown by a projection of five fluorescence intensity histograms of five different embryos. Scale bar: 100 µm and 50 µm, respectively. **d** Bean plots shows the distribution, means, and standard deviations of the sizes of e-cadherin-GFP clusters in the lateral and axial mesodermal plate measured in 20 WT and *pcdh18a* morphant embryos

To support the hypothesis that Pcdh18a increases trafficking of E-cad by endocytic recycling, an in vivo fluorescent recovery after bleaching (FRAP) of membrane-located E-cad-GFP was performed in zebrafish embryos at 5 hpf. We analysed three different bleach spots at the plasma membrane in five different embryos per treatment. We found that the FRAP into a bleached region of E-cad-GFP expressing membrane is reduced in a WT embryo compared to that in an embryo co-expressing E-cad-GFP and Pcdh18a (Fig. 4a). These data suggest that Pcdh18a facilitates recovery of fluorescence by E-cad-GFP, most likely by enhanced endocytic recycling to the membrane.

We then addressed which Pcdh18a domains are important for E-cad endocytosis. Full-length Pcdh18a is localized most prominently in vesicles (Fig. 4b). Pcdh18a lacking the intracellular domain (Pcdh18a-ECD) is enriched at the membrane independent of the presence of E-cad. Co-expression of WT-Pcdh18a together with Pcdh18a-ECD leads to co-internalization, suggesting that the WT form can interact with the ECD-mutant to induce uptake. Co-expression of E-cad with Pcdh18a leads to enhanced formation of intracellular E-cad-positive puncta, possibly, E-cad+ endocytic vesicles, whereas Pcdh18a-ECD is unable to route E-cad to endocytic vesicles. These experiments suggest that the extracellular domain of Pcdh18a is important for homophilic interactions, whereas the intracellular domain of Pcdh18a is important for internalization of Pcdh18a homodimers and Pcdh18a/E-cad heterodimers presumably used for recycling. In support of this hypothesis, co-expression of E-cad and Pcdh18a-ECD reduces recovery of E-cad-GFP after bleaching (Fig. 4a), suggesting that Pcdh18a-ECD reduces E-cad endocytosis and recycling. Protocadherins and cadherins also form dimers between neighbouring cells to regulate cell adhesion in a cluster (Perret et al. 2004). Similarly, we found that Pcdh18a also forms trans-interactions with E-cad, followed by endocytosis of the Pcdh18a/E-cad plaques (Supplementary Fig. S4c). In summary, we conclude that Pcdh18a enhances E-cad endocytosis and probably shifts the balance from degradation towards recycling. Indeed, our data suggest that Pcdh18a leads to an increase of E-cad/Rab-11 co-localization. This suggests an accumulation of E-cad in the recycling pathway, which may lead to an intracellular increase of E-cad eventually. Thus, our data suggest that Pcdh18a increases the E-cad levels in the membrane and in vesicles in the ppl cells.

Next, we analysed the function of endogenous Pcdh18a with respect to E-cad trafficking in the ppl. Subcellular E-cad concentrations were measured across the central ppl of *Tg(gsc:GFP)* transgenic zebrafish embryos. We discovered enhanced localization of E-cad-mCherry in the membrane and in vesicles in the ppl, as indicated by the peaks in the E-cad intensity histograms (Fig. 4c) and quantification of the number and sizes of E-cad clusters in the ppl (Fig. 4d). We did not observe these focal E-cad accumulations in the lpm cells surrounding the ppl or in the ppl of Pcdh18a-deficient embryos, suggesting that the ppl displays enhanced endocytosis of E-cad and we suggest that this is mediated by endogenous Pcdh18a.

### Recycling of E-cad clusters determine cell migration

**Fig. 5:**
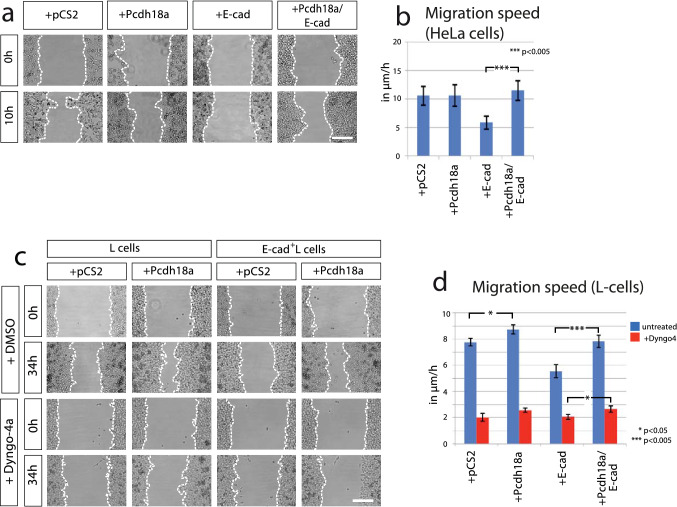
Pcdh18a affects E-cadherin dependent cell migration. **a** Wound-healing assay in HeLa cells. Cells were transfected with the indicated constructs and their migratory behaviour was monitored for 10 h after removing the insert. The dotted line shows limits of the confluent cell layer. **b** Quantification of the migration speed of HeLa cells. **c** Wound-healing assay in L cells. Cells were transfected with indicated constructs. After 24 h, L cells were treated with DMSO (1%) or Dyngo4a endocytosis inhibitor (1 µM in DMSO 1%). The migratory behaviour of cells was monitored in a time lapse for 34 h. **d** Quantification of the migration speed of L cells after blocking endocytosis. All wound-healing assays were conducted in independent triplicate, distances of the gap were measured at ten fixed positions. Mean values, SEM and significance are indicated (*p value < 0.05, ***p value < 0.005; unpaired Student’s *t* test). Scale bar: 200 µm

Cadherin-mediated cell adhesion complexes control cell migration in various contexts. Whereas an increase of cell adhesiveness usually leads to cell aggregation and reduced cell migration (Maître and Heisenberg 2013; Langhe et al. 2016), downregulation of E-cadherin in the epithelial–mesenchymal transition enhances disassembly of epithelial junction and subsequent malignant invasion of single cells (Scarpa et al. 2015). However, our experiments suggest that enhanced Pcdh18a/E-cad presentation at the plasma membrane can also increase migration of cell clusters. To resolve this discrepancy, we employed wound-healing assay in a controlled in vitro environment to analyse the function of the ppl key players in cell migration. First, we ectopically expressed Pcdh18a and E-cad in highly motile human cervical cancer cells (HeLa), which display low endogenous expression of E-cad (Hazan et al. 2004). We found no alteration in the migratory activity after transfection of Pcdh18a (Fig. 5a, b). Conversely, we observed that the motility of the E-cad-transfected HeLa cells was significantly decreased. Notably, we found that the expression of Pcdh18a in the E-cad-positive cells sped up cell migration to levels comparable to the control cells. In summary, we find that E-cad expression reduces migration of HeLa cells and Pcdh18a antagonizes the effect of E-cad. Together, with the data on co-localization (Fig. 3a) and endocytosis (Fig. 4), these observations suggest a functional interaction of Pcdh18a and E-cad influencing migratory cell behaviour.

Next, to measure cell motility with altered endocytic routing of E-cad, we set up additional wound-healing assay utilizing L cells. The transfection efficiency of Pcdh18a to L cells was about 40% (Supplementary Fig. S5a). L cells were then treated with Dyngo-4a, a blocker of dynamin-dependent endocytosis. Similar to our results with HeLa cells, we found that Pcdh18a expression increased cell migration in E-cad-GFP+ L cells (Fig. 5c, d). The reduction in endocytic trafficking of E-cad through the inhibition of dynamin function led to a significant decrease in cell migration, which could only partially be compensated by Pcdh18a expression. To provide further evidence that Pcdh18a acts functionally together with E-cad, we compared the migration speed of E-cad-GFP+ L cells transfected with Pcdh18a with E-cad-GFP+ L cells treated with the E-cad blocking antibody DECMA (Pece and Gutkind 2000). We found that both treatments increased migration speed of E-cad-GFP+ L cells in a similar way (Supplementary Fig. S5b). The in vitro experiments suggest that enhanced E-cad leads to decreased cell migration; however, Pcdh18a converts these slow migrating cells into fast migrating cells by enhancing E-cad recycling.

### Pcdh18a/E-cad-positive ppl cells can help notochord organization

**Fig. 6:**
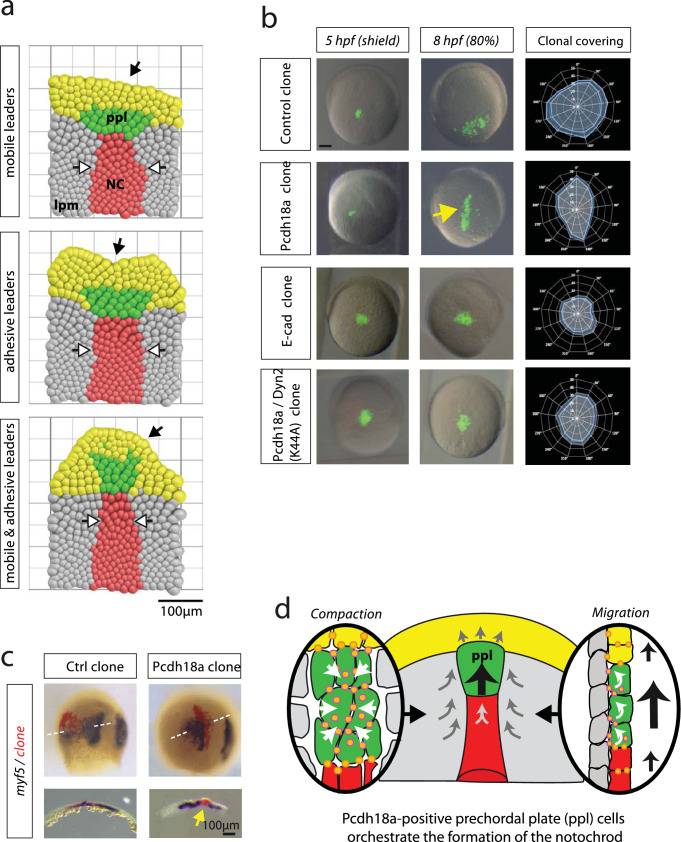
Directed cohort migration results in the formation of the rod-shaped notochord. **a** Visual rendering of the results of the simulations. See Supplementary Fig. S6 for the description of the cellular Potts model. **b** Embryos were microinjected with mRNAs for the indicated constructs (*pcdh18a*: 0.3 ng, *e-cad*: 0.4 ng, *dyn2K44A*: 0.2 ng). At 5 hpf, approximately 50 cells were grafted into the lateral embryonic margin of uninjected host embryos (*n* = 5). At 8 hpf, the migration and the directionality of the cell clusters were analysed. Animal pole was set to 0°, vegetal pole was set to 180°. Blue line indicates mean value of clonal coverage measured in ten different embryos per experiment and white lines indicate SEM. **c** Embryos from **b** were fixed and subjected to ISH for the lpm marker *myf5*. Horizontal cross sections revealed the formation of an ectopic rod-shaped structure of the Pcdh18a-positive clones in the lpm (yellow arrow). **d** Schematic summary of the function of the ppl in notochord morphogenesis. Pcdh18a/E-cadherin adhesion complexes (orange dots) increase cell adhesion within the ppl, leading to the cluster formation (left). In parallel, Pcdh18a controls endocytosis of E-cadherin adhesion complexes to allow fast cohort migration of the ppl cluster (right) to orchestrate intercalation of notochord cells. Scale bar: 100 µm

To study the self-organization of the mesodermal tissue during gastrulation, a lattice-based cellular Potts model (CPM) was implemented based on the Glazier–Graner–Hogeweg Model (Graner and Glazier 1992). This model recaptures our experimentally accessible cell properties and is defined by intercellular adhesion energies, intracellular surface and volume constraints, and migration properties (Fig. 6a; Supplementary Fig. S6). The CPM was used to address the question of which properties of individual cell groups were required to generate intercalation of the migrating cell layer into a notochord-like structure. In our modelling framework, every cell is represented in an object-oriented fashion by a physical location in the tissue, as well as cell type-dependent physical properties such as cell–cell adhesiveness, relative migration speed, and migration direction. We specified different cell types: the mesodermal leading edge, the ppl, the trailing notochord plate, and the surrounding lpm. Every cell, together with its interactions with its next neighbours, gives an energetic contribution to the total energy of the system, as specified in the Hamiltonian function of the CPM (see Methods). The system evolves from one state to the next by employing the Metropolis Monte-Carlo criterion for comparing these energetic contributions, giving rise to a statistically descriptive end state. The simulation started with cell ingression from the epiblast, forming the mesodermal cell layer. The first cells to appear were the E-cad-positive leading edge (Fig. 6a, yellow cells). These cells are an anteriorly migrating group of cells that are characterized by high cell–cell adhesiveness. Therefore, it is unlikely that these cells change neighbours. These cells were followed by the anteriorly migrating ppl (green cells) surrounded by lpm cells (grey cells). New lpm cells pushed into the field as the cells migrate forward. Simulations were stopped when the lpm reached an extension of 400 µm, which equates to 8 hpf. We studied three different scenarios for the ppl: mobile leaders, adhesive leaders, and mobile and adhesive leaders. Based on our observations of altered cell migration in vitro (Fig. 5), we increased the cell motility µ of the ppl by 100% compared to their neighbours (Fig. 6a, mobile leaders). We observed that the ppl was seen to exhibit an oblong shape that was perpendicular to the anteroposterior axis, similar to a droplet hitting a wall. This result suggests that the differential adhesion between the leading edge and the ppl, along with the weak cell–substrate interaction, inhibits the elongation of the notochordal plate (red cells, white arrows). The adhesion of the ppl was then increased without altering the cell migration speed (adhesive leaders). We observed that the trailing notochordal plate did not compact and showed a remarkably similar phenotype of embryos with reduced Pcdh18a levels (Fig. 1e). Interestingly, it seems that the ppl slows down the migration of the medial leading-edge cells, leading to a concave tissue shape (black arrow). Finally, we increased both the migration speed and adhesion (Fig. 6a, mobile and adhesive leaders). We found that the shape of the tissue changed: the E-cad-positive leading edge formed a convex outline and thus resembled the curved shape of the ppl in vivo. Even more striking than changes to the tissue shape, we observed that the central Pcdh18a-positive ppl and the notochordal plate condensed to a rod-shaped structure. We, therefore, hypothesize that the increased adhesiveness and migration speed of the ppl cells are essential factors to control the extension of notochord structure. As the CPM is nearest neighbour based, we conclude that the stretching forces generated from the ppl primarily operate on the trailing notochordal plate and secondarily on the adjacent lpm cells, remodelling the shape of individual cells as observed in the previous phenotypical analysis (Fig. 1).

Thus far, our experiments and simulations suggest that Pcdh18a is required to organize the shape of the ppl, which influences the shape of the notochord either by regulating gene expression in the notochord or by controlling cellular mechanics. In a test of the first hypothesis, we found that Pcdh18a-positive clones did not induce ectopic expression of the notochord marker *ntl* in the lpm (Supplementary Fig. S7a), in contrast to Chordin/Dkk1 positive clones, which generate a secondary Spemann organizer followed by the induction of a secondary notochord. Next, we performed a deep mRNA sequencing analysis of Pcdh18a-overexpressing embryos and Pcdh18a-morphant embryos. In concordance with our results, we did not detect alteration in the expression of mesodermal genes such as *chordin, gsc, ntl, shha*, and *shhb*, or in the expression of *e-cad* (Supplementary Fig. S7b).

During collective cell migration, the Rac1-mediated polarization of leader cells, the interaction of leader and follower cells and the migration process with retrograde flow of the adherens junctions, has been studied during oogenesis in *Drosophila*, lateral line formation in zebrafish, neural crest formation in *Xenopus,* and cancer invasion (Mayor and Etienne-Manneville 2016). Rac1 function—in addition to Wnt/PCP and E-cadherin—has also be elucidated during mesoderm morphogenesis in zebrafish (Dumortier et al. 2012; Dumortier and David 2015). However, the molecular mechanism regulating increased adhesiveness and fast migration of the very same cluster on a substrate—i.e., neighbouring cells—at the same time is not well understood. Therefore, we tested the second hypothesis: whether the Pcdh18a-positive clones determine tissue shape by regulating cohesive cell migration. Cells were grafted from the lateral blastoderm margin from a Pcdh18a-positive donor into the lateral blastoderm margin of a WT host at 5 hpf. The cells formed a compact and elongated cell cluster, which migrated towards the animal pole (Fig. 6b). The re-organization into rod-shaped ppl was even more obvious in horizontal sections of embryos subjected to in situ hybridization for the lpm marker *myf5* (Fig. 6c). We then asked whether this phenotype could be explained by enhanced E-cad localization at the cell membranes of the cluster cells, as suggested previously (Fig. 3a, b). E-cad-expressing cell clones were indeed observed in clusters (Fig. 6b); however, the shape of these clusters was circular, and the clustered cells migrated slowly towards the animal pole, similar to the in vitro wound-healing approach (Fig. 5) and the simulation ‘adhesive leaders’ (Fig. 6a). Indeed, analysis of E-cad localization during zebrafish epiboly suggests that endocytic E-cad treadmilling allowed the blastoderm cells to dynamically remodel their cell–cell contacts, leading to enhanced mobility (Song et al. 2013). To test our hypothesis whether Pcdh18a-regulated E-cad endocytosis promoted cohesion and migration, we grafted cells into a WT host embryo that expressed Pcdh18a and a mutated form of Dynamin2^K44A^ to block endocytosis (Fig. 6b). We found that Pcdh18a/Dyn2^K44A^-positive cells form a cluster. However, similar to the E-cad-positive cell cluster, they rarely migrated, which is in concordance with the Dyngo-4 treatment to inhibit endocytosis of E-cad-positive L cells (Fig. 5c). Based on these results, we conclude that Pcdh18a regulates directed cohort migration behaviour of the ppl. We suggest that this process serves as an equivalent to the ppl during axial mesoderm morphogenesis.

## Discussion

Here, we identified Pcdh18a as a regulator of mesoderm organization in zebrafish. Pcdh18a function has been analysed in embryonic development in fish previously (Aamar and Dawid 2008). Pcdh18a functions in promoting cell–cell adhesion during epiboly movement; however, a molecular mechanism for Pcdh18a function in the mesoderm is unclear. We analysed Pcdh18a function during morphogenesis of the notochord. Based on our analysis, we define three consecutive steps in zebrafish notogenesis (Fig. 6d). The first involuting cells are the E-cad-positive, leading marginal edge cells of the the mesodermal plate. Collective and directional movement towards the anterior pole is intrinsically regulated by E-cad and Wnt-PCP signalling (Ulrich et al. 2005; Dumortier et al. 2012). The ppl organizes in an elongated cell cluster and moves anteriorly due to a cadherin-mediated pulling force from the posterior side (Weber et al. 2012). Second, we describe the ppl as an axial mesodermal cell population, which forms an elongated cell cluster after ingression (Fig. 6d). In mice, a similar compaction of dispersed mesodermal cells located anterior to the forming Spemann organizer was observed in the early gastrula and these cells give rise to the anterior part of the notochord (Yamanaka et al. 2007). We observed that the E-cadherin-positive ppl marched towards the animal pole as a cohesive cell group during gastrulation (Ulrich et al. 2005; Blanco et al. 2007). Thus, the ppl pushes forward, leading to the formation of a convexly curved mesodermal cell sheet. A prerequisite to exert these pushing forces is a faster migration of the ppl with regard to the surrounding tissue, which we observe (Figs. 2, 6). There is supporting evidence that the ppl in *Xenopus* and zebrafish migrates also faster than the adjacent mesodermal cells to exert axial extension (Hara et al. 2013; Glickman et al. 2003). Third, the ppl physically instructs the notochord plate cells, to intercalate and elongate. We conclude that this process supports the condensation of the trailing notochord.

Convergent extension in the axial mesoderm during zebrafish gastrulation is driven mainly by mediolateral intercalation (Glickman et al. 2003; Yin et al. 2008; Williams et al. 2018). Furthermore, the notochordal plate cells actively migrate towards the animal pole and thereby extend their shape in the anteroposterior direction (Keller 2002, 2005). Indeed, time-lapse analysis in zebrafish revealed that the notochordal plate lengthens rapidly during gastrulation (Glickman et al. 2003). The blockage of Pcdh18a function (Figs. 1, 2) and our tissue model (Fig. 6a) indicate that the inhibition of ppl migration results in reduced condensation of the notochordal plate and slowdown of its extension. Furthermore, WT ppl can rescue the phenotype in Pcdh18a-deficient embryos (Fig. 2e, f) and clonal expression of Pcdh18a lead to the formation of an ectopic rod-shaped ppl in the lpm (Fig. 6b, c). Therefore, we argue that in addition to the suggested intrinsic capacity of the notochord cells to intercalate and elongate, the ppl migration influences the elongation of the notochordal plate. The cell behaviour of the notochordal plate cells and the ppl migration lead to the elongation, alignment, and intercalation of the notochord cells. Pcdh18a-positive ppl cells could either exert physical forces on the notochordal plate or present a signal for the notochord cells to direct the cellular rearrangement in the trailing central mesoderm. Recent work in *Xenopus* has demonstrated that cells can polarize in response to traction exerted by their neighbours through cadherins (Weber et al. 2012). Furthermore, our simulations supports this notion by suggesting that the condensation of the notochord is independent of the movement of the lpm cells. We find that convergent extension of the notochord is less important for lpm formation (Figs. 2, 6). Consistent with this, embryos with compromised convergence movements of the lpm do not necessarily also exhibit notochord extension defects (Bakkers et al. 2004). This is also supported by migration analysis in mice, in which no obvious movements in the lpm were observed (Ybot-Gonzalez et al. 2007; Wang et al. 2006), and axial notochord progenitors still undergo convergent extension movements to trigger notochord elongation by cell intercalations (Yamanaka et al. 2007; Yen et al. 2009).

How does Pcdh18a facilitate the migration of the ppl? The δ2 protocadherin subfamily comprises five members—Pcdh8, Pcdh10, Pcdh17, Pcdh18 and Pcdh19, which are involved in tissue migration and morphogenesis (Hayashi and Takeichi 2015). Knockdown of *pcdh18a* by using morpholino oligonucleotides causes impaired cell movements (Aamar and Dawid 2008), and knockdown of *pcdh19* results in impaired convergence during neurulation (Emond et al. 2009). Pcdh8 (also known as PAPC) is expressed in the paraxial mesoderm and is involved in cell sorting and convergent extension during gastrulation (Chal et al. 2017; Kim et al. 1998; Medina et al. 2004; Unterseher et al. 2004). Furthermore, protocadherins can act in concert with cadherins to modulate cell adhesion: Pcdh8 acts together with C-cadherin and PCP components in cell migration (Kraft et al. 2012; Chen and Gumbiner 2006), also with N-cadherin to promote its endocytosis in neuron (Yasuda et al. 2007). This notion is in accordance with previous results suggesting that prechordal plate migration is a collective E-cadherin, Wnt/PCP and Rac1-dependent process (Ulrich et al. 2005; Dumortier et al. 2012). The ppl cells require a directional signal provided by contact to the endogenous plate. It has been suggested that directionality of ppl is influenced by Wnt/PCP signalling (Ulrich et al. 2003), Fgf signalling (Shimada et al. 2008), and PDGF signalling (Nagel et al. 2004; Kai et al. 2008). Our observations suggest that similarly Pcdh18a influences cell migration of the ppl by interaction with E-cad. We suggest that Pcdh18a facilitates cycling of E-cad between the plasma membrane and recycling endosomes (Fig. 3), which allows ppl migration (Figs. 5, 6). Endocytosis and recycling of E-cadherin is a prerequisite for proper remodelling of cell junctions to allow cell intercalation and tissue remodelling (Brüser and Bogdan 2017). Several studies in cell culture and in *Drosophila* tissues show that E-cadherin is principally transported to Rab5-positive early endosomes and Rab11-positive recycling endosomes, which is crucial in tissue remodelling and collective cell migration (Brüser and Bogdan 2017). In concert, we observe an increase of E-cad in Rab5/11-positive endosomes after activation of Pcdh18a (Fig. 3). Furthermore, Pcdhs—including Pcdh18a—can accelerate cohort tissue movement by actin cytoskeleton reorganization through the WAVE complex, specifically Nap1 (Chen et al. 2014; Biswas et al. 2014; Nakao et al. 2008), which could be a further process acting downstream of the observed phenotype.

Finally, cadherin adhesion clusters respond to mechanical pulling forces by eliciting a strain-stiffening response (le Duc et al. 2010). Therefore, it is tempting to speculate that the induction of tension on the forming notochord by the ppl leads to a reinforcement of an axial structural scaffold. Our data suggest that Pcdh18a-mediated E-cad turnover in the ppl cells generates forces that are required for the organization of the mesoderm and generation of the hallmark of Chordata—the notochord.

## Electronic supplementary material

Below is the link to the electronic supplementary material.

Supplementary file1. Supplementary Figure S1: Whole-mount in situ hybridization (ISH) of zebrafish embryos. **a**–**g** Mapping of expression patterns of indicated genes in the mesodermal plate at 6.5 h post-fertilisation (hpf) and 8.5 hpf. Asterisks mark prechordal plate cells (ppl). **h** Specificity of the *pcdh18a* MO in zebrafish embryos. Embryos were microinjected in the one-cell stage with Pcdh18a-GFP mRNA and control MO or MO against *pcdh18a*. Fluorescent images show example embryos at 5 hpf. **i** Embryos were injected with 0.5 mM control MO and *pcdh18a* MO, raised to 9 hpf and subjected for ISH for the indicated markers. Arrows mark the width of the notochord and the length of the anteroposterior axis. **j** *pcdh18a* morphant embryos display severe malformations in the trunk and tail area at 24 hpf (arrows), whereas the head seems unaltered. Analysis of *Tg(shh:GFP) *embryos also revealed a severe bending in Pcdh18a deficient embryos (arrows). Scale bar: 100 μm (EPS 39895 kb)

Supplementary file2. Supplementary Figure S2: Analysis of zygotic and maternal zygotic *pcdh18a* mutant embryos. **a** The single guide RNA (sgRNA) target sequence for *pcdh18a* mutagenesis and the location of 5 bp deletion in the mutant. **b** Dorsal images of wild type (WT), *pcdh18a* zygotic and maternal zygotic (MZ) mutant embryos at 32 h post-fertilisation (hpf). Zygotic and MZ mutant embryos showed normal tail elongation. **c** Notochord extension in WT, zygotic and MZ *pcdh18a* mutant embryos marked by *ntl* expression at 9 hpf. **d** Width of the notochord was not significantly altered in *pcdh18a* mutant embryos, MZ *pcdh18a* mutant embryos, and in MZ *pcdh18a* mutant embryos injected with the MO against *pcdh18a*. Statistical test was performed by one-way ANOVA (EPS 15182 kb)

Supplementary file3. Supplementary Figure S3: Phenotypic analysis of zebrafish embryos. **a**–**d** 3D-optic flow analysis. **a**, **b** Examples of one more wild-type (WT) and *pcdh18a* MO embryo—colour code and nomenclature same as Fig. 2b, c. **c** The relative velocity (ectoderm velocity–mesoderm velocity) in the *ϕ* direction (same colour coding as Fig. 2c). **d** The transverse extension rate of the mesoderm for a WT and *pcdh18a* MO embryo averaged over 5–8 hpf. Positive values correspond to broadening of the mesoderm perpendicular to the migration axis. Negative values correspond to thinning of the mesoderm perpendicular to the migration axis (EPS 2036 kb)

Supplementary file4. Supplementary Figure S4: Subcellular localization of Pcdh18a and its influence on E-cadherin. **a** Confocal images of live zebrafish embryos at 5 hpf of indicated markers. **b** L cells stably expressing E-cad-GFP were transfected with Pcdh18a-mCherry and fixed, then stained with DAPI. Arrows show the co-localization of E-cad-GFP with Pcdh18a-mCherry. Scale bar: 10 μm. **c** Live imaging of neighbouring cell clones expressing Pcdh18a-GFP and a blue memCFP or E-cad-GFP/memCFP and Pcdh18a-mCherry/memCFP. At the 8-cell stage, the embryos were injected with 0.1 ng of pcdh18a-mCherry/memCFP mRNAs in one blastomere and e-cad-GFP or pcdh18a-GFP mRNA in the adjacent blastomere. Trans-internalization of Pcdh18a/Pcdh18a and Pcdh18a/E-cad was observed (yellow arrows). Inset shows co-localization at higher magnification. Scale bar: 10 µm (EPS 43834 kb)

Supplementary file5. Supplementary Figure S5: Analysis of transfection rate and migratory behaviour of L cells. **a** FACS analysis of L cells transfected with E-cad-GFP and Pcdh18a-mCherry. Transfection efficiency of L cells was measured using fluorescence activated cell sorting (FACS). M1 represents auto-fluorescence, M2 represents fluorescence of transfected constructs. **b** Quantification of the migration speed of L cells after blocking E-cad function with E-cad-blocking antibody (DECMA-1). The migratory behaviour of the cells was monitored in time lapse for 12 h. The experiments were conducted in independent duplicates. Mean values, SEM and significance by Student *t* test are indicated (EPS 1423 kb)

Supplementary file6. Supplementary Figure S6: Cellular potts model (CPM) for tissue dynamics in the mesoderm. **a** Schematic representation of the simulation protocol using three cells. (1) First, a random lattice site at the surface of a cell is chosen (x). (2) Next, either empty medium (M) or a random source (s) is chosen within two lattice sites (bounded area) of the initial target site (red). The Hamiltonian for the hypothetical new state is calculated and evaluated within the bounded area and the difference *ΔH* computed. (3) Depending on the probability P from state *γ* to state *γ*' one of the two possible outcomes is chosen, either the source lattice site is copied into the target, or the target remains the same. **b** Definition for the Hamiltonian *H* and modifications to *ΔH* for our CPM. Exact parameter values are found in **h**. (1) The Hamiltonian for state *γ* consists of three sums and defines the total energy of the system. The first sum is running over each cell σ at a lattice site $\overrightarrow{x}$ and its neighbouring lattice sites *x*′. This sum represents the energetic contributions by the cellular adhesiveness *J*, which is dependent on the cellular types $\tau \left( {\sigma \left( {\vec{x}} \right)} \right)$ and $\tau \left( {\sigma \left( {\vec{x^{\prime}}} \right)} \right)$. For the exact values of *J*, see **g**. The Kronecker-delta *δ* makes sure that only different cells are contributing, and self-interactions are excluded. The second and third sum are running over each cell and sum up volume and surface contributions scaled by a factor *λ*_*V*_, respectively *λ*_*S*_. Each cell tries to preserve its original volume *V*_0_ and surface *S*_0_. (2) We defined a modification *ΔH*_*M*_ to introduce mobility into our simulations with a linear anterior-posterior potential *c*. This modification is coupled to the mobility *μ* of the source cell at the neighbouring lattice site *x*′ for the hypothetical state *γ*' and the mobility constant *λ*_*M*_. See **a** for a visual explanation of the simulation scheme. The total change in *ΔH* is obtained from both contributions of *ΔH*_*B*_ = *H*(*γ*') − *H*(*γ*) and *ΔH*_*M*_. **c** The hypothetical state *γ*' is accepted with a probability given by the Metropolis criterion. **d**–**f** Simulation results, where every pixel represents one lattice site. *M* (white), leading edge (yellow), ppl (green), lpm (grey), and NC (red). We indicate in the top right corner of each simulation the number of Monte Carlo steps (MCs). In each Monte Carlo step, we loop through each surface pixel of every cell. The grid scale is 50 μm. **d** For the case of mobile leaders we see that no curving of the leading edge takes place. The ppl takes an oblong shape perpendicular to the direction of movement. The NC keeps a broad shape. **e** In the case of adhesive leaders, we see that the ppl still keeps an oblong shape. A dip occurs in the leading edge, resulting from the high attraction of the ppl. **f** In the case of mobile and adhesive leaders we see a strong curvature of the leading edge. The ppl and NC take a sharp and long configuration. **g** Values for adhesiveness as used in the simulation in matrix representation for the case of mobile leaders and non-mobile leaders. **h** Parameters as used in the simulation and for the definition of the Hamiltonian. We use a system in reduced units, where the cell motility is reduced to 1. The only free parameters are cell adhesiveness and mobility. The adhesiveness was chosen in such a way, that adhesive leaders stick roughly twice as strong together as non-adhesive leader (EPS 7233 kb)

Supplementary Figure S7 (PDF 6698 kb)

## References

[CR1] Aamar E, Dawid IB (2008). Protocadherin-18a has a role in cell adhesion, behavior and migration in zebrafish development. Dev Biol.

[CR2] Bakkers J, Kramer C, Pothof J, Quaedvlieg NE, Spaink HP, Hammerschmidt M (2004). Has2 is required upstream of Rac1 to govern dorsal migration of lateral cells during zebrafish gastrulation. Development.

[CR3] Biswas S, Emond MR, Duy PQ, Hao LT, Beattie CE, Jontes JD (2014). Protocadherin-18b interacts with Nap1 to control motor axon growth and arborization in zebrafish. Mol Biol Cell.

[CR4] Blanco MJ, Barrallo-Gimeno A, Acloque H, Reyes AE, Tada M, Allende ML, Mayor R, Nieto MA (2007). Snail1a and Snail1b cooperate in the anterior migration of the axial mesendoderm in the zebrafish embryo. Development.

[CR5] Brand M, Granato M, Nüsslein-Volhard C, Nüsslein-Volhard C, Dahm R (2002). Keeping and raising zebrafish. Zebrafish: a practical approach.

[CR6] Brasch J, Harrison OJ, Honig B, Shapiro L (2012). Thinking outside the cell: how cadherins drive adhesion. Trends Cell Biol.

[CR7] Brüser L, Bogdan S (2017). Adherens junctions on the move-membrane trafficking of E-cadherin. Cold Spring Harb Perspect Biol.

[CR8] Burger A, Lindsay H, Felker A, Hess C, Anders C, Chiavacci E, Zaugg J, Weber LM, Catena R, Jinek M, Robinson MD, Mosimann C (2016). Maximizing mutagenesis with solubilized CRISPR-Cas9 ribonucleoprotein complexes. Development.

[CR9] Chal J, Guillot C, Pourquié O (2017). PAPC couples the segmentation clock to somite morphogenesis by regulating N-cadherin-dependent adhesion. Development.

[CR11] Chen X, Gumbiner BM (2006). Paraxial protocadherin mediates cell sorting and tissue morphogenesis by regulating C-cadherin adhesion activity. J Cell Biol.

[CR10] Chen B, Brinkmann K, Chen Z, Pak CW, Liao Y, Shi S, Henry L, Grishin NV, Bogdan S, Rosen MK (2014). The WAVE regulatory complex links diverse receptors to the actin cytoskeleton. Cell.

[CR12] Clark BS, Winter M, Cohen AR, Link BA (2011). Generation of Rab-based transgenic lines for in vivo studies of endosome biology in zebrafish. Dev Dyn.

[CR13] Dumortier JG, David NB (2015). The TORC2 component, Sin1, controls migration of anterior mesendoderm during zebrafish gastrulation. PLoS ONE.

[CR14] Dumortier JG, Martin S, Meyer D, Rosa FM, David NB (2012). Collective mesendoderm migration relies on an intrinsic directionality signal transmitted through cell contacts. Proc Natl Acad Sci USA.

[CR16] El-Brolosy MA, Stainier DYR (2017). Genetic compensation: a phenomenon in search of mechanisms. PLoS Genet.

[CR15] El-Brolosy KZ, Rossi A, Kuenne C, Günther S, Fukuda N, Kikhi K, Boezio GLM, Takacs CM, Lai SL, Fukuda R, Gerri C, Giraldez AJ, Stainier DYR (2019). Genetic compensation triggered by mutant mRNA degradation. Nature.

[CR17] Emond MR, Biswas S, Jontes JD (2009). Protocadherin-19 is essential for early steps in brain morphogenesis. Dev Biol.

[CR18] Feldman B, Gates MA, Egan ES, Dougan ST, Rennebeck G, Sirotkin HI, Schier AF, Talbot WS (1998). Zebrafish organizer development and germ-layer formation require nodal-related signals. Nature.

[CR19] Fichtner D, Lorenz B, Engin S, Deichmann C, Oelkers M, Janshoff A, Menke A, Wedlich D, Franz CM (2014). Covalent and density-controlled surface immobilization of E-cadherin for adhesion force spectroscopy. PLoS ONE.

[CR20] Glickman NS, Kimmel CB, Jones MA, Adams RJ (2003). Shaping the zebrafish notochord. Development.

[CR21] Graner F, Glazier J (1992). Simulation of biological cell sorting using a two-dimensional extended Potts model. Phys Rev Lett.

[CR22] Hara Y, Nagayama K, Yamamoto TS, Matsumoto T, Suzuki M, Ueno N (2013). Directional migration of leading-edge mesoderm generates physical forces: implication in Xenopus notochord formation during gastrulation. Dev Biol.

[CR24] Hayashi S, Takeichi M (2015). Emerging roles of protocadherins: from self-avoidance to enhancement of motility. J Cell Sci.

[CR23] Hayashi S, Inoue Y, Kiyonari H, Abe T, Misaki K, Moriguchi H, Tanaka Y, Takeichi M (2014). Protocadherin-17 mediates collective axon extension by recruiting actin regulator complexes to interaxonal contacts. Dev Cell.

[CR25] Hazan RB, Qiao R, Keren R, Badano I, Suyama K (2004). Cadherin switch in tumor progression. Ann NY Acad Sci.

[CR26] Heisenberg C-P, Solnica-Krezel L (2008). Back and forth between cell fate specification and movement during vertebrate gastrulation. Curr Opin Genet Dev.

[CR27] Heisenberg CP, Tada M, Rauch GJ, Saúde L, Concha ML, Geisler R, Stemple DL, Smith JC, Wilson SW (2000). Silberblick/Wnt11 mediates convergent extension movements during zebrafish gastrulation. Nature.

[CR28] Hwang WY, Fu Y, Reyon D, Maeder ML, Tsai SQ, Sander JD, Peterson RT, Yeh JR, Joung JK (2013). Efficient genome editing in zebrafish using a CRISPR-Cas system. Nat Biotechnol.

[CR29] Kai M, Heisenberg CP, Tada M (2008). Sphingosine-1-phosphate receptors regulate individual cell behaviours underlying the directed migration of prechordal plate progenitor cells during zebrafish gastrulation. Development.

[CR30] Keller R (2002). Shaping the vertebrate body plan by polarized embryonic cell movements. Science.

[CR31] Keller R (2005). Cell migration during gastrulation. Curr Opin Cell Biol.

[CR32] Kim SH, Yamamoto A, Bouwmeester T, Agius E, Robertis EM (1998). The role of paraxial protocadherin in selective adhesion and cell movements of the mesoderm during Xenopus gastrulation. Development.

[CR33] Kimelman D, Griffin KJ (2000). Vertebrate mesendoderm induction and patterning. Curr Opin Genet Dev.

[CR34] Kraft B, Berger CD, Wallkamm V, Steinbeisser H, Wedlich D (2012). Wnt-11 and Fz7 reduce cell adhesion in convergent extension by sequestration of PAPC and C-cadherin. J Cell Biol.

[CR35] Langhe RP, Gudzenko T, Bachmann M, Becker SF, Gonnermann C, Winter C, Abbruzzese G, Alfandari D, Kratzer MC, Franz CM, Kashef J (2016). Cadherin-11 localizes to focal adhesions and promotes cell-substrate adhesion. Nat Commun.

[CR36] le Duc Q, Shi Q, Blonk I, Sonnenberg A, Wang N, Leckband D, de Rooji J (2010). Vinculin potentiates E-cadherin mechanosensing and is recruited to actin-anchored sites within adherens junctions in a myosin II-dependent manner. J Cell Biol.

[CR37] Maître J-L, Heisenberg C-P (2013). Three functions of cadherins in cell adhesion. Curr Biol.

[CR38] Mattes B, Weber S, Peres J, Chen Q, Davidson G, Houart C, Scholpp S (2012). Wnt3 and Wnt3a are required for induction of the mid-diencephalic organizer in the caudal forebrain. Neural Dev.

[CR39] Mayor R, Etienne-Manneville S (2016). The front and rear of collective cell migration. Nat Rev Mol Cell Biol.

[CR40] Medina A, Swain RK, Kuerner KM, Steinbeisser H (2004). Xenopus paraxial protocadherin has signaling functions and is involved in tissue separation. EMBO J.

[CR41] Myers DC, Sepich DS, Solnica-Krezel L (2002). Convergence and extension in vertebrate gastrulae: cell movements according to or in search of identity?. Trends Genet.

[CR42] Nagel M, Tahinci E, Symes K, Winklbauer R (2004). Guidance of mesoderm cell migration in the Xenopus gastrula requires PDGF signaling. Development.

[CR43] Nakao S, Platek A, Hirano S, Takeichi M (2008). Contact-dependent promotion of cell migration by the OL-protocadherin-Nap1 interaction. J Cell Biol.

[CR44] Pece S, Gutkind JS (2000). Signaling from E-cadherins to the MAPK pathway by the recruitment and activation of epidermal growth factor receptors upon cell-cell contact formation. J Biol Chem.

[CR45] Perret E, Leung A, Feracci H, Evans E (2004). Trans-bonded pairs of E-cadherin exhibit a remarkable hierarchy of mechanical strengths. Proc Natl Acad Sci USA.

[CR46] Rossi A, Kontarakis Z, Gerri C, Nolte H, Hölper S, Krüger M, Stainier DY (2015). Genetic compensation induced by deleterious mutations but not gene knockdowns. Nature.

[CR47] Roszko I, Sawada A, Solnica-Krezel L (2009). Regulation of convergence and extension movements during vertebrate gastrulation by the Wnt/PCP pathway. Semin Cell Dev Biol.

[CR48] Roszko I, Sepich D, Jessen JR, Chandrasekhar A, Solnica-Krezel L (2015). A dynamic intracellular distribution of Vangl2 accompanies cell polarization during zebrafish gastrulation. Development.

[CR49] Sampath K, Rubinstein AL, Cheng AM, Liang JO, Fekany K, Solnica-Krezel L, Korzh V, Halpern ME, Wright CV (1998). Induction of the zebrafish ventral brain and floorplate requires cyclops/nodal signalling. Nature.

[CR50] Scarpa E, Szabó A, Bibonne A, Theveneau E, Parsons M, Mayor R (2015). Cadherin switch during EMT in neural crest cells leads to contact inhibition of locomotion via repolarization of forces. Dev Cell.

[CR51] Schier AF, Neuhauss SC, Helde KA, Talbot WS, Driever W (1997). The one-eyed pinhead gene functions in mesoderm and endoderm formation in zebrafish and interacts with no tail. Development.

[CR52] Scholpp S, Brand M (2003). Integrity of the midbrain region is required to maintain the diencephalic-mesencephalic boundary in zebrafish no isthmus/pax2.1 mutants. Dev Dyn.

[CR53] Shimada A, Yabusaki M, Niwa H, Yokoi H, Hatta K, Kobayashi D, Takeda H (2008). Maternal-zygotic medaka mutants for fgfr1 reveal its essential role in the migration of the axial mesoderm but not the lateral mesoderm. Development.

[CR54] Shkumatava A, Fischer S, Müller F, Strähle U, Neumann CJ (2004). Sonic hedgehog, secreted by amacrine cells, acts as a short-range signal to direct differentiation and lamination in the zebrafish retina. Development.

[CR55] Shih J, Keller R (1992). Cell motility driving mediolateral intercalation in explants of Xenopus laevis. Development.

[CR56] Song S, Eckerle S, Onichtchouk D, Marrs JA, Nitschke R, Driever W (2013). Pou5f1-dependent EGF expression controls E-cadherin endocytosis, cell adhesion, and zebrafish epiboly movements. Dev Cell.

[CR57] Stemmer M, Thumberger T, Del Sol KM, Wittbrodt J, Mateo JL (2015). CCTop: An intuitive, flexible and reliable CRISPR/Cas9 target prediction tool. PLoS ONE.

[CR58] Stemple DL (2005). Structure and function of the notochord: an essential organ for chordate development. Development.

[CR59] Tada M, Heisenberg C-P (2012). Convergent extension: using collective cell migration and cell intercalation to shape embryos. Development.

[CR60] Theveneau E, Mayor R (2012). Cadherins in collective cell migration of mesenchymal cells. Curr Opin Cell Biol.

[CR61] Ulrich F, Concha ML, Heid PJ, Voss E, Witzel S, Roehl H, Tada M, Wilson SW, Adams RJ, Soll DR, Heisenberg CP (2003). Slb/Wnt11 controls hypoblast cell migration and morphogenesis at the onset of zebrafish gastrulation. Development.

[CR62] Ulrich F, Krieg M, Schötz EM, Link V, Castanon I, Schnabel V, Taubenberger A, Mueller D, Puech PH, Heisenberg CP (2005). Wnt11 functions in gastrulation by controlling cell cohesion through Rab5c and E-cadherin. Dev Cell.

[CR63] Unterseher F, Hefele JA, Giehl K, De Robertis EM, Wedlich D, Schambony A (2004). Paraxial protocadherin coordinates cell polarity during convergent extension via Rho A and JNK. EMBO J.

[CR64] Vig DK, Hamby AE, Wolgemuth CW (2016). On the quantification of cellular velocity fields. Biophys J.

[CR65] Wang J, Hamblet NS, Mark S, Dickinson ME, Brinkman BC, Segil N, Fraser SE, Chen P, Wallingford JB, Wynshaw-Boris A (2006). Dishevelled genes mediate a conserved mammalian PCP pathway to regulate convergent extension during neurulation. Development.

[CR66] Warga RM, Kimmel CB (1990). Cell movements during epiboly and gastrulation in zebrafish. Development.

[CR67] Weber GF, Bjerke MA, DeSimone DW (2012). A mechanoresponsive cadherin-keratin complex directs polarized protrusive behavior and collective cell migration. Dev Cell.

[CR68] Williams MLK, Sawada A, Budine T, Yin C, Gontarz P, Solnica-Krezel L (2018). Gon4l regulates notochord boundary formation and cell polarity underlying axis extension by repressing adhesion genes. Nat Commun.

[CR69] Yamanaka Y, Tamplin OJ, Beckers A, Gossler A, Rossant J (2007). Live imaging and genetic analysis of mouse notochord formation reveals regional morphogenetic mechanisms. Dev Cell.

[CR70] Yasuda S, Tanaka H, Sugiura H, Okamura K, Sakaguchi T, Tran U, Takemiya T, Mizoguchi A, Yagita Y, Sakurai T, De Robertis EM, Yamagata K (2007). Activity-induced protocadherin arcadlin regulates dendritic spine number by triggering N-cadherin endocytosis via TAO2beta and p38 MAP kinases. Neuron.

[CR71] Ybot-Gonzalez P, Savery D, Gerrelli D, Signore M, Mitchell CE, Faux CH, Greene ND, Copp AJ (2007). Convergent extension, planar-cell-polarity signalling and initiation of mouse neural tube closure. Development.

[CR72] Yen WW, Williams M, Periasamy A, Conaway M, Burdsal C, Keller R, Lu X, Sutherland A (2009). PTK7 is essential for polarized cell motility and convergent extension during mouse gastrulation. Development.

[CR73] Yin C, Kiskowski M, Pouille PA, Farge E, Solnica-Krezel L (2008). Cooperation of polarized cell intercalations drives convergence and extension of presomitic mesoderm during zebrafish gastrulation. J Cell Biol.

