# Supplementary figures and images for: Pcdh18a regulates endocytosis of E-cadherin during axial mesoderm development in zebrafish
Source: Histochem Cell Biol. 2020 Jun 1;154(5):463–80. doi: 10.1007/s00418-020-01887-5 (PMC7609436; doi:10.1007/s00418-020-01887-5)

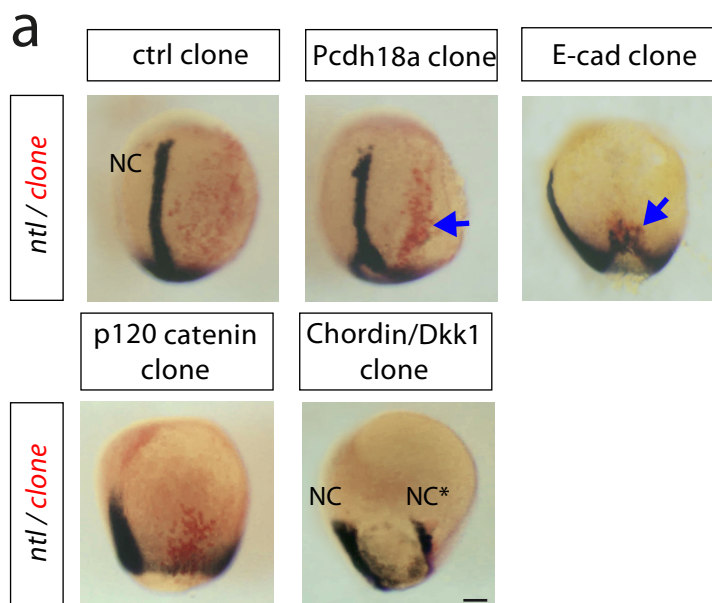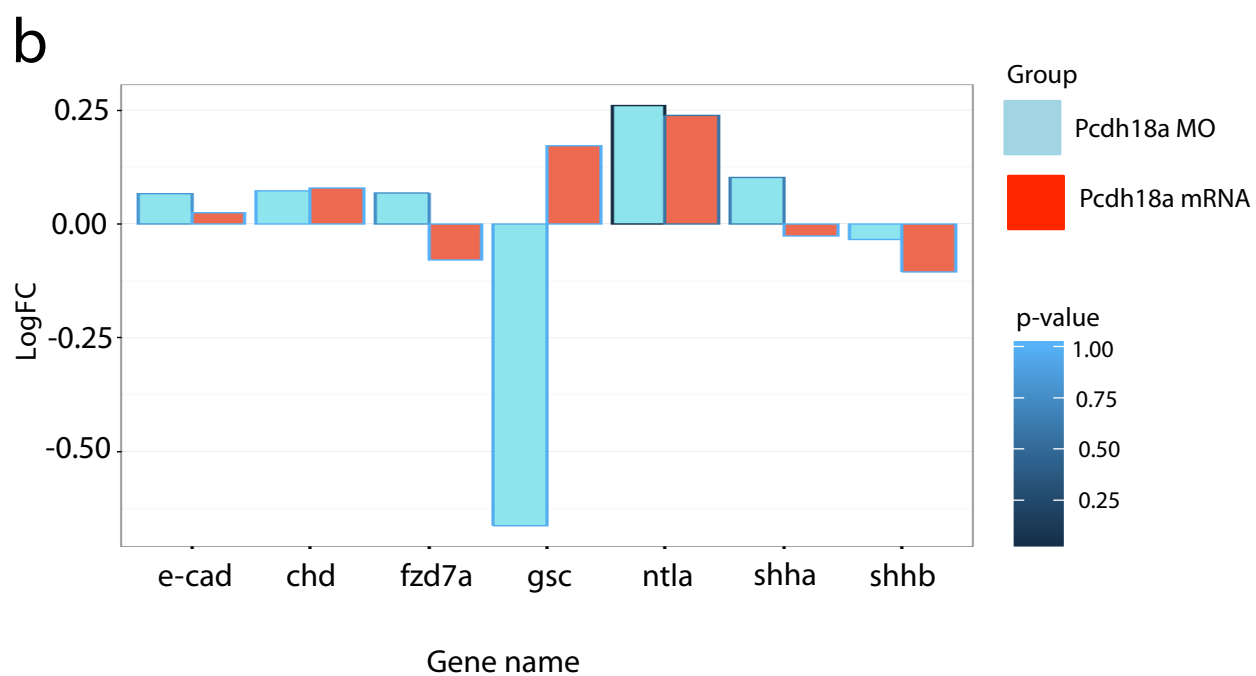

Supplementary Figure 7, Bosze et al., 2020

Supplement: Supplementary file 7 — Supplementary Figure S7 (PDF 6698 kb) [file 418_2020_1887_MOESM7_ESM.pdf]
